# Vibration Error Compensation of LiDAR Imaging with the Aiding of INS for Precise Navigation

**DOI:** 10.3390/s26134277

**Published:** 2026-07-05

**Authors:** Songlai Han, Tanjie Chen, Jing Dong, Xudong Yu, Xuesong Liu

**Affiliations:** 1 Institute of Aerospace Science and Technology, Central South University, Changsha 410012, China; songlai.han@csu.edu.cn (S.H.); 245801003@csu.edu.cn (T.C.); hotsjsvt@163.com (X.L.); 2 College of Advanced Interdisciplinary Studies, National University of Defense Technology, Changsha 410073, China; yuxudong07@nudt.edu.cn

**Keywords:** error compensation, inertial navigation, kalman filters, laser radar

## Abstract

Point cloud images from LiDAR often suffer distortion due to platform vibration. This paper proposes a LiDAR-INS (Inertial Navigation System) integrated navigation method to address the challenge of low positioning accuracy in complex environments. To solve problems like GPS signal denial and vibration interference, we present a method for achieving centimeter-level positioning. This method uses INS attitude information to compensate for LiDAR vibration errors. A vibration error model is established to quantify the impact of vibration on point cloud distortion. High-frequency INS attitude data is then used to correct the LiDAR point cloud distortion caused by platform vibration. Leveraging the non-repetitive scanning pattern of prism-based LiDAR, a joint compensation strategy for vibration error and angular error is proposed. This strategy enhances both point cloud density and positioning robustness. State and observation equations for the LiDAR-INS integrated navigation system are derived. A Kalman filter is employed to achieve optimal data fusion between the LiDAR and INS. Finally, field experiments were conducted in both laboratory settings and a typical application scenario: a tunnel construction site. These experiments validate the effectiveness of the proposed method.

## 1. Introduction

Mining excavation, subway construction, and coal mining operations are frequently conducted in underground tunnels. These operations often require navigation and positioning technology for assistance, demanding high positioning accuracy and stability. Tunneling operations, in particular, require centimeter-level positioning accuracy and angular minute-level orientation accuracy [1]. Tunnel environments are characterized by being narrow, confined, and irregularly shaped. GPS signals are unavailable underground, lighting and visual conditions are poor, and the electromagnetic environment is complex. These harsh conditions degrade the accuracy and stability of various sensors. The specific environmental constraints of tunnels impose significant limitations on navigation. Accurate navigation is crucial for improving operational efficiency and ensuring safety in underground environments. Therefore, researching navigation methods for narrow, elongated underground tunnels is of significant importance.

Tunnel navigation represents a typical indoor navigation scenario. Commonly employed indoor navigation and positioning technologies include UWB, Wi-Fi, ZigBee, SLAM, visual-inertial SLAM (viSLAM), inertial navigation, among others [2,3,4,5]. Among these, UWB positioning technology offers high precision, resistance to multipath fading, and strong anti-interference capabilities [6]; however, it requires deploying base stations within the tunnel and exhibits lower positioning accuracy in narrow, elongated areas [7]. Wi-Fi positioning technology utilizes wireless local area networks formed by access points to achieve positioning in complex environments, featuring simple equipment and wide coverage; yet, its accuracy depends on device density, and signals are prone to environmental obstruction within tunnels [8]. ZigBee positioning technology, characterized as a low-power, low-bandwidth radio method with stable transmission, sees limited application in tunnel operations due to its restricted transmission range and relatively low positioning accuracy [9]. SLAM technology acquires and analyzes environmental image data via cameras or other sensors for positioning; nevertheless, tunnels frequently suffer from poor or absent lighting, and significant machinery-induced vibrations can degrade imaging quality [10]. viSLAM, extending visual SLAM by fusing IMU computational results [11], enhances robustness and effectively resolves scale initialization issues; however, it is susceptible to drift during operation, involves cumbersome calibration procedures, and has a constrained measurement range [12]. Related work includes Wang [13] compensating vibration based on LiDAR’s inherent characteristics, Hu [14] investigating camera-based calibration for LiDAR, and Chen [15] fusing high-frequency wheel odometry measurements with Normal Distribution Transform (NDT) registration to correct errors via a two-stage compensation framework.

Inertial navigation is a navigation technology based on the principle of inertial measurement, which calculates changes in position and orientation by measuring acceleration and angular velocity [16]. The INS primarily consists of gyroscopes and accelerometers. It establishes a navigation coordinate system based on the output of the gyroscopes and then computes navigation information for the vehicle in the inertial reference frame by combining the output from the accelerometers. This is an autonomous navigation technique; it neither relies on external information nor emits energy to determine the vehicle’s attitude, velocity, and position. Primarily applied in fields such as petroleum drilling, engineering surveying, submarines, and unmanned aerial vehicles (UAVs) [17,18,19], it offers high accuracy and excellent real-time performance. However, due to error accumulation, prolonged navigation can lead to position drift, often necessitating correction from other positioning methods to mitigate errors [20].

Previous studies address related but distinct problems. Wang et al. [13] considered signal-level vibration compensation for coherent FMCW LiDAR; Hu et al. [14] focused on LiDAR-camera extrinsic calibration in tunnel mapping; Chen et al. [15] used wheel odometry and NDT registration for motion-distortion correction; and Xiao et al. [21] introduced vehicle-dynamics constraints into LiDAR-inertial odometry. In contrast, the present study focuses on point-wise motion-distortion compensation for a non-repetitive prism-based direct-detection LiDAR and on retroreflective-marker-based positioning in narrow, GNSS-denied tunnels.

The specific contributions are as follows: (1) a vibration-induced point-cloud error model is formulated using the relative pose variation between the acquisition time of each LiDAR point and the selected frame reference time; (2) high-rate INS pose estimates and calibrated LiDAR-INS extrinsic parameters are used for point-wise deskewing of the non-repetitive prism scan; (3) retroreflective-target observations are integrated with an error-state INS model to bound inertial drift; and (4) laboratory and tunnel experiments are used to evaluate point-cloud compactness and positioning accuracy. The novelty is therefore the integration of point-level INS-aided deskewing, prism-LiDAR target localization, and error-state fusion for the specific constraints of narrow tunnel navigation, rather than LiDAR-INS fusion alone.

## 2. Vibration Error Modell of LiDAR Imaging with INS Position and Attitude Feedback

### 2.1. LiDAR Positioning Approach Based on Imaging and Recognition

LiDAR operates within its field of view (FoV) by employing a laser modulator to periodically emit laser pulses. These pulses reflect off targets and are received by a photodetector. The distance to the target is calculated based on the time delay of the reflected light. Rotation scanning is then used to acquire the target’s three-dimensional position and shape information. Multiple scans are merged to generate high-precision 3D images. As a novel type of non-repetitive scanning LiDAR, prism-based LiDAR exhibits increasing point cloud density over time. Furthermore, by adjusting the rotational speeds of its two prisms, it generates non-repetitive scanning paths. Given sufficient time, the scanned point cloud covers the entire FoV.

This study employs a prism-based Livox Mid-40 LiDAR (Livox Technology Company Limited, Shenzhen, China) for positioning. Its non-repetitive scanning pattern increases the spatial coverage within the circular field of view (FoV) as the integration time increases, which is useful for observing retroreflective targets in a narrow tunnel. According to the manufacturer’s user manual, the nominal range precision is 2 cm (1σ at 20 m, measured using an 80–reflectivity target at 25 °C), and the angular accuracy is less than 0.1° [22]. These are sensor specifications obtained under controlled conditions and should not be interpreted as the accuracy of the complete navigation system. In the present work, the Mid-40 is used to identify retroreflective targets and support centimeter-scale target-center estimation and positioning. Millimeter-scale crack detection is outside the scope of this study.

**Figure 1:**
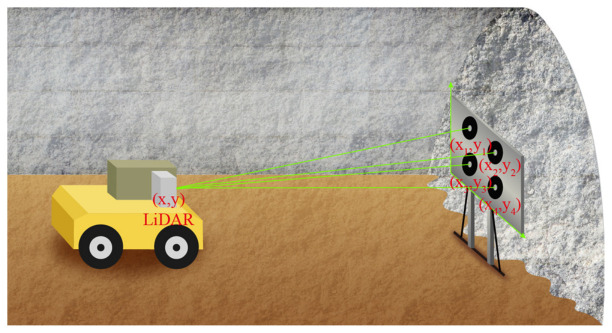
Schematic diagram of identification and positioning solution.

The identification and positioning scheme is implemented as follows: Four retroreflective targets are deployed within the tunnel. The central sticker on each target generates strong laser retroreflection, making them highly conspicuous to the LiDAR. By leveraging distance measurements between these targets and the LiDAR, the operating machinery can be accurately positioned, as shown in the Figure 1 following.

The LiDAR scan generates a 3D point cloud of the environment. The central area of the retroreflective targets exhibits significantly higher reflectivity than surrounding surfaces. Using reflective intensity as the threshold, a filtering algorithm extracts the point cloud corresponding to the target centers. Since the LiDAR may not directly face the targets orthogonally, the extracted point clouds are mathematically fitted to elliptical models to calculate the targets’ central coordinates. The specific procedures are as follows:

(a) The high-reflectivity point cluster associated with each retroreflective target is extracted using the intensity threshold described above. A local target plane is estimated from the 3D cluster, and the points are orthogonally projected onto this plane. The resulting two-dimensional coordinates are denoted by $\left(x_{i},y_{i}\right),\;i=1,\ldots ,N$, where N is the number of points retained for ellipse fitting.
(b) The projected target boundary is represented by the general conic equation:

(1) $${{Ax}_{i}}^{2}+Bx_{i}y_{i}+{{Cy}_{i}}^{2}+Dx_{i}+Ey_{i}+F=0,\;i=1,\ldots ,N$$

where $\beta_{c}={\left[A,B,C,D,E,F\right]}^{T}$ is the conic-parameter vector. The condition B^2^ − 4AC < 0 ensures that the fitted conic is an ellipse.

(c) The ellipse parameters are estimated using the constrained direct least-squares method:

(2) $$\hat{\beta_{c}}=\arg min\sum_{i=1}^{N}{\left(m_{i}^{T}\beta_{c}\right)}^{2},\;subject\;to\;4AC-B^{2}=1$$

In the formula, $\hat{\beta_{c}}$ represent the ellipse parameter vector estimated using the constrained least-squares method. $4AC-B^{2}=1$ represent the ellipse constraint used to ensure that the fitted conic is an ellipse and to avoid the trivial all-zero solution.

(3) $$m_{i}={\left[{x_{i}}^{2},x_{i}y_{i},{y_{i}}^{2},x_{i},y_{i},1\right]}^{T}$$

The constraint removes the arbitrary scale of $\beta_{c}$ and prevents the least-squares solution from degenerating into a non-elliptic conic.

(d) After $\hat{\beta_{c}}$ has been obtained, the ellipse center in the local target plane is calculated independently of the semi-axis lengths as follows:

(4) $$\left[\begin{array}{c} x_{c}\\ y_{c} \end{array}\right]={-\left[\begin{array}{cc} 2A & B\\ B & 2C \end{array}\right]}^{-1}\left[\begin{array}{c} D\\ E \end{array}\right]$$

The center is then transformed from the local target plane back to the LiDAR coordinate frame. Only the center coordinates are required for the subsequent positioning calculation; the semi-major axis, semi-minor axis, and orientation can be recovered from the conic parameters if needed.

(e) The constrained least-squares ellipse fitting was evaluated over 2000 LiDAR frames on a computer equipped with an Intel Core i7-12700H processor. The mean fitting time was 0.83ms per target, and the maximum fitting time was 2.71 ms. Depending on the target range and incidence angle, the number (N) of retained points used for each ellipse fit ranged from 58 to 246. These results indicate that the fixed-size generalized eigenvalue problem can be solved within the LiDAR frame period.

The raw LiDAR observations are modeled in the native range–angle domain so that systematic bias and random noise are treated separately:

(5) $$r_{m}=r+b_{r}+n_{r},\;\alpha_{m}=\alpha +b_{\alpha }+n_{\alpha },\;\beta_{m}=\beta +b_{\beta }+n_{\beta }$$

Here, $b_{r}$, $b_{\alpha }$, and $b_{\beta }$ denote the systematic range, elevation-angle, and azimuth-angle biases, whereas $n_{r}$, $n_{\alpha }$, and $n_{\beta }$ denote zero-mean random measurement noise. The systematic terms are determined by an offline calibration using retroreflective targets whose coordinates are independently measured by the total station. The calibrated biases are removed before target center estimation, while the residual random uncertainty is propagated to Cartesian coordinates through the measurement Jacobian and included in the measurement covariance $R_{k}$.

The point-processing chain consists of an intensity gate, a physically valid range gate, neighborhood-based outlier rejection, projection onto the local target plane, and constrained ellipse fitting. The retroreflective target points were extracted using a fixed intensity threshold of 180, and only points within a range of 2–30 m were retained. Statistical outlier rejection was then performed using 20 nearest neighbors and a standard-deviation multiplier of 2.0. The remaining points were projected onto the estimated local target plane before constrained ellipse fitting. Offline LiDAR calibration was conducted using retroreflective targets located at surveyed distances of 5, 10, 20, and 30 m. At each distance, 300 repeated target observations were collected. The estimated systematic biases were $b_{r}$ = +1.2 cm for range, $b_{\alpha }$ = −0.28° for elevation, and $b_{\beta }$ = +0.24° for azimuth. After applying these corrections, the residual range and angular biases were below 0.6 cm and 0.06°, respectively.

LiDAR ranging does not require visible-light illumination and is therefore suitable for low-light or dark tunnel environments. However, its performance is not completely invariant to environmental conditions: strong background irradiance, target reflectivity, incidence angle, surface material, and range can affect return intensity, valid-point rate, and measurement variance. Accordingly, the advantages claimed here are limited to three-dimensional active ranging and low-light operability, rather than complete insensitivity to illumination. These characteristics are supported by prior tunnel and prism-LiDAR studies [14,22] and the manufacturer’s specifications [23]. The illumination robustness protocol used in this work is described in Section 4.5.

### 2.2. LiDAR Vibration Compensation Based on INS Attitude Feedback

#### 2.2.1. Impact Analysis of Vibration on LiDAR Precision Positioning

**Figure 2:**
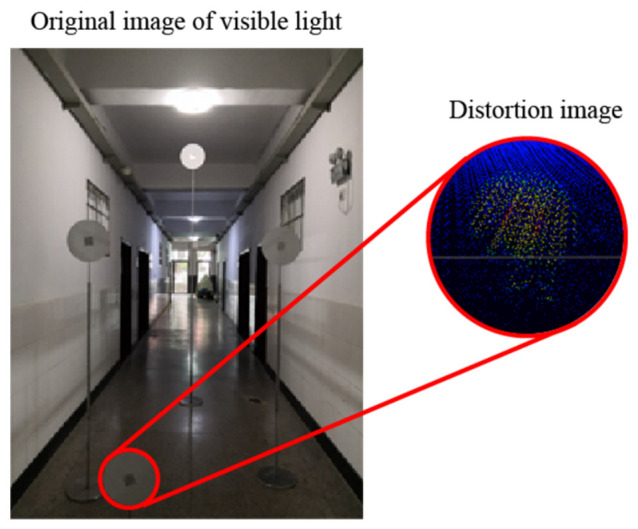
Distortion plot of a point cloud image.

Within narrow underground tunnels, uneven ground surfaces induce significant platform vibration and sudden rotational angle changes during motion. These dynamics exacerbate point cloud distortion, which subsequently causes blurred target imaging. This blurring hinders accurate extraction of target center points, degrades positioning precision, and may even lead to complete localization failure. Therefore, compensating for LiDAR vibration-induced point cloud distortion prior to feature extraction is critical. During real-time localization, LiDAR moves with the platform and experiences operational vibrations. Consequently, acquired point clouds inevitably exhibit distortion, as illustrated in Figure 2.

Point cloud distortion errors are primarily induced by vibration, manifesting as positional deviations of point centroids within the global coordinate frame. These deviations compromise measurement accuracy. To quantify distortion errors, we establish the kinematic model of the platform below. Vibration components include translational displacement and rotational attitude deviation.

Error-model coordinate frames are defined as follows: W is the world/reference frame, I is the IMU body frame fixed to the IMU sensor axes, and L is the LiDAR measurement frame. All three frames are right-handed.

Ideal State Coordinate Formulation (No vibration):

(6) $$q_{world,ideal}=R_{0}(t)L_{meas}+P_{0}(t)$$

In the formula, $R_{0}(t)\in \;\mathrm{SO}(3)$ is the ideal rotation matrix from the LiDAR frame L to the world frame $W,P_{0}(t)\in \;{\mathbb{R}}^{3}$ is the ideal LiDAR-origin position expressed in W, $L_{meas}\in \;{\mathbb{R}}^{3}$ is the measured point coordinate expressed in L, and $q_{world,ideal}\in \;{\mathbb{R}}^{3}$ is the corresponding ideal point coordinate expressed in W.

Under operational conditions, the residual translational perturbation $\delta P(t)\in \;{\mathbb{R}}^{3}$ and the small attitude-error vector $\delta \theta (t)\in \;{\mathbb{R}}^{3}$ are modeled as zero-mean first-order Gauss–Markov processes. The two processes are assumed mutually independent at the same time, but each process is temporally correlated. Their scalar isotropic correlation models are $R_{p}(\Delta t)=\sigma_{p}^{2}\exp\left(-\frac{\left|\Delta t\right|}{\tau_{p}}\right)$, $R_{\theta }(\Delta t)=\sigma_{p}^{2}\exp\left(-\frac{\left|\Delta t\right|}{\tau_{\theta }}\right)$, where $\tau_{p}$ and $\tau_{\theta }$ are the translational and angular correlation times. The residual translational and angular perturbations were treated as zero-mean random variables for the instantaneous single-frame error analysis. Their standard deviations, estimated from the detrended vibration data, were $\sigma_{p}=6.5$ mm and $\sigma_{\theta }=0.035$°, respectively. Temporal correlation parameters were not estimated in the present experiment; therefore, the following RMSE expression characterizes the instantaneous distortion uncertainty rather than the complete time-domain vibration process.

(7) $$q_{world,actual}\approx R_{0}(t)(I+[\delta R(t)]\times )L_{meas}+P_{0}(t)+\delta P(t)$$

In the formula, [δR(t)]× denotes the 3 × 3 skew-symmetric matrix formed from the small attitude-error vector δR(t).

The positioning error can be expressed as follows:

(8) $$\delta q=q_{world,actual}-q_{world,ideal}\approx R_{0}(t)([\delta R(t)]\times )q_{meas}+\delta P(t)$$

The magnitude of the instantaneous distortion error is quantified by the Euclidean norm ${\left\|\delta q\right\|}_{2}$. For the single-time covariance propagation used below, translational and angular perturbations are assumed isotropic and mutually independent; their temporal correlations are described by the preceding Gauss–Markov model and do not change the same-time variance expression.

(9) $$\mathrm{Var}(\delta q)\approx \sigma_{p}^{2}+\sigma_{r}^{2}d^{2}$$

In the formula, $d=\left\|L_{meas}\right\|$ denotes the point-to-LiDAR distance, $\sigma_{p}$ represents the standard deviation of positional vibration, and $\sigma_{r}$ signifies the standard deviation of angular vibration. The root mean square error (RMSE) of distortion can be expressed as follows:

(10) $$\mathrm{RMSE}\approx \sqrt{\sigma_{p}^{2}+\sigma_{r}^{2}d^{2}}$$

This formulation demonstrates that the positioning error term $\sigma_{p}^{2}$ induced by positional vibration is distance-independent, while the error term $\sigma_{r}^{2}d^{2}$ caused by angular vibration scales proportionally to the square of the distance d. Consequently, the positioning error is significantly amplified for distant points.

#### 2.2.2. LiDAR Vibration Error Compensation Method

The inertial measurement unit (IMU) provides raw angular-rate and specific-force measurements, whereas the strapdown inertial navigation system (INS) processes these measurements to produce attitude, velocity, and position. In the remainder of this paper, “IMU” denotes the sensing unit and its raw measurements, and “INS” denotes the complete navigation solution.

The gyroscope and accelerometer measurement models are written as:

(11) $$\omega_{m}\left(t\right)=\omega \left(t\right)+b_{g}\left(t\right)+n_{g}(t)$$

(12) $$f_{m}\left(t\right)=f\left(t\right)+b_{a}\left(t\right)+n_{a}(t)$$

where $\omega_{m}$ and $f_{m}$ are the gyroscope and accelerometer outputs, $b_{g}$ and $b_{a}$ are the gyroscope and accelerometer biases, and $n_{g}$ and $n_{a}$ are zero-mean measurement noises. Noise realizations are unknown and cannot be subtracted directly. The INS mechanization therefore uses $\hat{\omega }=\omega_{m}-{\hat{b}}_{g}$ and $\hat{f}=f_{m}-{\hat{b}}_{a}$, where ${\hat{b}}_{g}$ and ${\hat{b}}_{a}$ are obtained from the initial static calibration and are subsequently updated by the Kalman filter. The residual random noises are represented in the process-noise covariance matrix Q.

Let $T_{L}^{W}\left(t\right)\in SE(3)$ denote the LiDAR pose in the world frame at time t, with rotation $R_{L}^{W}\left(t\right)\in SO(3)$ and position $p_{L}^{W}\left(t\right)\in R^{3}$. It is obtained from the high-rate INS pose and the calibrated rigid LiDAR–IMU extrinsic transformation $T_{L}^{I}=(R_{L}^{I},p_{L}^{I})$. The symbol p is used for translation to avoid confusion with the time variable t. INS poses are interpolated to the timestamp of each LiDAR point; attitude is interpolated on SO(3), and position is interpolated from the INS position and velocity solution.

For a LiDAR point $P^{L_{t}}$ acquired at time t within a frame whose reference time is t_0_, the relative transformation is:

(13) $$T_{L_{t}}^{L_{t_{0}}}={\left[T_{L}^{W}\left(t_{0}\right)\right]}^{-1}T_{L}^{W}(t)$$

and the point deskewed to the start-of-frame coordinate system is:

(14) $$P^{L_{t_{0}}}=R_{L_{t}}^{L_{t_{0}}}P^{L_{t}}+P_{L_{t}}^{L_{t_{0}}}$$

Equation (13) compensates both rotational and translational motion during the scan. It avoids directly double-integrating uncorrected accelerometer measurements for each point and ensures that the bias estimates used for deskewing are consistent with the INS/Kalman-filter solution.

Two critical factors affecting measurement accuracy must be addressed: 1. Angular bias inherent in LiDAR encoder factory calibration inevitably compromises angular measurement precision; 2. Temporal synchronization challenges arise due to disparate operating principles between sensors-the IMU computes platform velocity, position, and attitude through acceleration/angular rate integration, while the LiDAR acquires environmental data through rotational scanning where each motor revolution generates one point cloud frame (frame rate equaling rotations per second). Independent hardware clocks create temporal misalignment between these asynchronous data streams.

The angular correction is implemented through the explicit spherical-to-Cartesian measurement Jacobian. For $d={\left[\sigma ,\xi ,l\right]}^{T}$ and $p^{L}(d)={\left[l\cos\sigma \cos\xi ,l\cos\sigma \sin\xi ,l\sin\sigma \right]}^{T}$, define $J_{d}=\frac{\partial P^{L}}{\partial d}$. The full 3 × 3 matrix is written explicitly in Equation (31) (denoted there by $C_{j}$). For an offline-calibrated angular error $\delta \theta_{L}={\left[\delta \sigma ,\delta \xi \right]}^{T}$, the correction uses the first two columns $J_{\theta }$ of $J_{d}$, so that the corrected point is ${\hat{P}}^{L}=P^{L}+J_{\theta }\delta \theta_{L}$. This makes the dimensions and the relationship between the angular calibration and the Cartesian correction explicit.

(15) $$\hat{P}(L_{t})=P\left(L_{t}\right)+J_{r}\cdot \delta \theta$$

The parameter Δt denotes the residual constant clock offset between the LiDAR and IMU timestamps during one experimental run; it is a software time-alignment parameter rather than hardware-level synchronization. Before each run, Δt is estimated offline by shifting the two data streams and minimizing the relative-rotation discrepancy between LiDAR-derived frame-to-frame motion and the integrated IMU angular-rate sequence. The corrected LiDAR timestamp is $\hat{t}=t+\Delta t$, and the estimated offset is held constant during that run.

## 3. Optimal Fusion Approach of INS and LiDAR for Precise Tunnel Navigation

### 3.1. Overall Scheme

**Figure 3:**
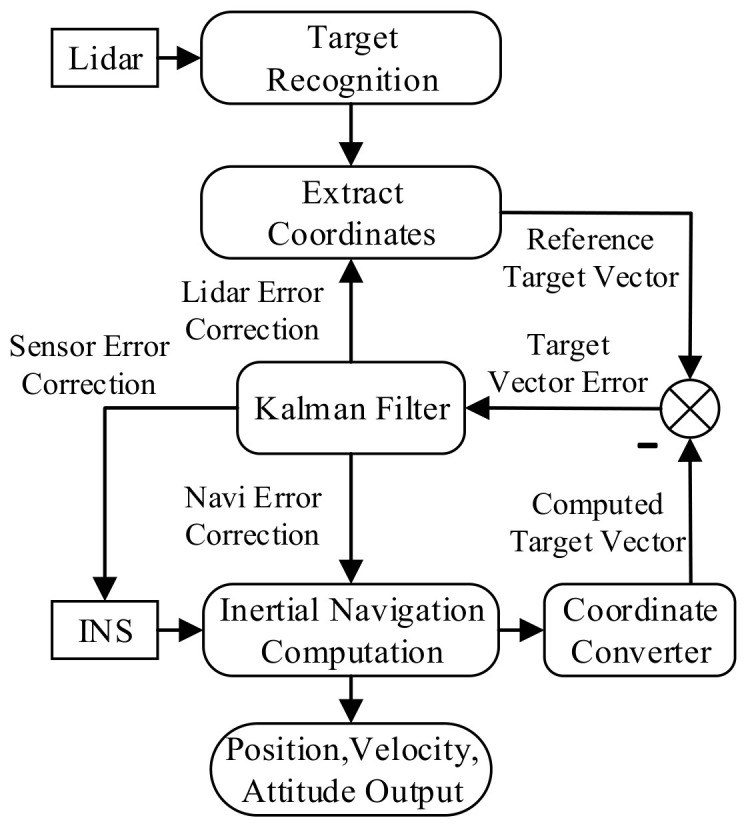
INS and LiDAR integrated navigation algorithm.

The data fusion architecture between LiDAR and the INS is illustrated in Figure 3. To address feature-deficient environments in tunnels [22], retroreflective targets must be pre-deployed for LiDAR identification prior to navigation operations, with their coordinates preloaded into the system. While inertial navigation operates independently without external inputs, its positioning error accumulates rapidly during prolonged navigation. To mitigate this limitation, a LiDAR-INSintegrated navigation scheme is implemented: LiDAR actively measures pre-deployed fiducial markers, thereby correcting accumulated INS drift and enhancing overall navigation accuracy.

The computational procedure for this integrated navigation algorithm comprises:

I. Observation error acquisition: During LiDAR-INS data fusion, the position vector of fiducial markers is obtained either directly via LiDAR measurement or computationally through INS using reference coordinates. The position vector discrepancy is derived from the difference between these two calculation methods.
II. Data fusion filtering: Estimated INS and LiDAR errors are corrected through filtering. High-frequency INS outputs subsequently compensate LiDAR observations, yielding the final fused navigation solution.

### 3.2. Dynamics for Optimal Estimation

The INS error equations adopt the ψ-angle error model [24], as shown in Equations (16)–(18):

(16) $$\delta {\dot{r}}^{n}=\delta V^{n}-\omega_{en}^{n}\times \delta r^{n}$$

(17) $$\delta {\dot{V}}^{n}=-\left(\omega_{in}^{n}{+\omega }_{ie}^{n}\right)\times \delta V^{n}-\psi^{n}\times f^{n}+C_{b}^{n}\nabla^{b}$$

(18) $${\dot{\psi }}^{n}=-\omega_{in}^{n}\times \psi^{n}+C_{b}^{n}\varepsilon^{b}$$

Superscripts and subscripts denote coordinate frames. The n-frame is the local-level North–East–Down (NED) navigation frame; the b-frame is the IMU body frame fixed to the IMU sensor axes; the l-frame is the LiDAR measurement frame; the i-frame is the Earth-centered inertial frame; and the e-frame is the Earth-centered Earth-fixed frame. All frames are right-handed. The vehicle frame is not assumed identical to the b-frame unless the calibrated mounting rotation is applied. The vectors $\delta r^{n}$, $\delta V^{n}$, and $\psi^{n}$ are position, velocity, and small attitude errors expressed in n. The specific force is measured by the accelerometers in the b-frame and is denoted $f^{b}$; $C_{b}^{n}$ transforms vectors from b to n. The bias notation $b_{a}$ and $b_{g}$ and the noise notation $n_{a}$ and $n_{g}$ are used consistently throughout the manuscript.

IMU bias and drift compensation are implemented in two stages. First, with the platform stationary, the initial gyroscope bias is obtained from the mean angular-rate output and the initial accelerometer bias is obtained after removing the gravity projection predicted from the initialized attitude. Second, $b_{a}$ and $b_{g}$ are included as estimated states and modeled as slowly varying random processes. After each LiDAR update, the estimated navigation, bias, and residual LiDAR-bias errors are fed back to the nominal INS solution and the error state is reset. Zero-mean LiDAR measurement noise is not included as a state; it is represented by $R_{k}$, while $b_{L}$ represents only residual systematic target-coordinate bias.

The system equation of the Kalman filter is based on the error equation, expressed as follows:

The continuous error-state model is $\dot{x}=Fx+Gw$, where w contains accelerometer and gyroscope white noise, accelerometer- and gyroscope-bias driving noise, and residual LiDAR-bias driving noise. For a sufficiently small IMU interval Δt, the discrete covariance is approximated by $Q_{c}\approx GQ_{c}G^{T}\Delta t$; matrix-exponential discretization is used when a larger propagation interval is adopted. $Q_{c}$ is determined from the sensor data sheet and/or Allan-deviation fitting, rather than by arbitrary trial-and-error tuning.

The measurement model is $z_{k}=H_{k}x_{k}+v_{k}$, with $E\left[v_{k}v_{k}^{T}\right]=R_{k}$ is obtained from repeated static observations of the retroreflective targets after bias correction. When native LiDAR uncertainties are specified in elevation, azimuth, and range, their covariance is propagated to Cartesian coordinates by $j_{d}$ and then rotated into the navigation frame. A normalized-innovation-squared gate is used to reject target observations inconsistent with the assumed covariance.

The initial covariance P_0_ is diagonal or block diagonal, with entries equal to the squared uncertainty of the initialized position, velocity, attitude, accelerometer bias, gyroscope bias, and residual LiDAR bias. The adopted values and their determination methods are summarized in Table 1.

The form of the system matrix F in the equation is given by the following equation:

(19) $$F=\left[\begin{array}{cccccc} F_{11} & F_{12} & 0 & 0 & 0 & 0\\ 0 & F_{22} & F_{23} & F_{24} & 0 & 0\\ 0 & 0 & F_{33} & 0 & F_{35} & 0\\ 0 & 0 & 0 & 0 & 0 & 0\\ 0 & 0 & 0 & 0 & 0 & 0\\ 0 & 0 & 0 & 0 & 0 & 0 \end{array}\right]$$

(20) $$F_{11}=\left[\begin{array}{ccc} 0 & \omega_{enU}^{n} & -\omega_{enN}^{n}\\ -\omega_{enU}^{n} & 0 & \omega_{enE}^{n}\\ \omega_{enN}^{n} & -\omega_{enE}^{n} & 0 \end{array}\right]$$

(21) $$F_{12}=I_{3\times 3}$$

(22) $$F_{22}=\left[\begin{array}{ccc} 0 & 2\omega_{ieU}^{n}+\omega_{enU}^{n} & -2\omega_{ieN}^{n}+\omega_{enN}^{n}\\ -2\omega_{ieU}^{n}+\omega_{enU}^{n} & 0 & 2\omega_{ieE}^{n}+\omega_{enE}^{n}\\ 2\omega_{ieN}^{n}+\omega_{enN}^{n} & -2\omega_{ieE}^{n}+\omega_{enE}^{n} & 0 \end{array}\right]$$

(23) $$F_{23}=\left[\begin{array}{ccc} 0 & -f_{U}^{n} & f_{N}^{n}\\ f_{U}^{n} & 0 & -f_{E}^{n}\\ -f_{N}^{n} & f_{E}^{n} & 0 \end{array}\right]$$

(24) $$F_{24}=C_{b}^{n}$$

(25) $$F_{33}=\left[\begin{array}{ccc} 0 & \omega_{inU}^{n} & -\omega_{inN}^{n}\\ -\omega_{inU}^{n} & 0 & \omega_{inE}^{n}\\ \omega_{inN}^{n} & -\omega_{inE}^{n} & 0 \end{array}\right]$$

(26) $$F_{35}=-C_{b}^{n}$$

### 3.3. Measurements for Optimal Estimation

#### 3.3.1. The Landmark Positions Measured by the LiDAR

The landmark j measured by the LiDAR has native coordinates $d_{j}={\left[\sigma_{j},\xi_{j},l_{j}\right]}^{T}$, where $\sigma_{j}$ is elevation, $\xi_{j}$ is azimuth, and $l_{j}$ is range. Its Cartesian coordinate $v_{j}^{l}$ is obtained by the spherical-to-Cartesian mapping. For a small native measurement error $\delta d_{j}$, the Cartesian perturbation is $\delta v_{j}^{l}=J_{j}\delta d_{j}$, where $J_{j}\equiv \frac{\partial v_{j}^{l}}{\partial d_{j}}$. The matrix written explicitly in Equation (28) is this Jacobian.

(27) $$\delta v_{j}^{l}=C_{1}\delta d$$

(28) $$C_{1}=\left[\begin{array}{ccc} -l_{j}\sin\sigma_{j}\cos\xi_{j} & -l_{j}\cos\sigma_{j}\sin\xi_{j} & \cos\sigma_{j}\cos\xi_{j}\\ -l_{j}\sin\sigma_{j}\sin\xi_{j} & l_{j}\cos\sigma_{j}\cos\xi_{j} & \cos\sigma_{j}\sin\xi_{j}\\ l_{j}\cos\sigma_{j} & 0 & \sin\sigma_{j} \end{array}\right]$$

The LiDAR measurements are projected into the n-coordinate system as follows:

(29) $$v_{j}^{n}=C_{b}^{n}C_{l}^{b}v_{j}^{l}$$

In the above formula, $C_{l}^{b}$ is the calibrated rotation from the LiDAR frame to the IMU body frame, and $C_{b}^{n}$ is the INS attitude matrix from the IMU body frame to the NED navigation frame. Here, “body frame” means the IMU sensor-axis frame; any vehicle-to-IMU mounting rotation is included in the calibrated transformation. The lever arm between LiDAR and IMU origins is handled separately in the position equation.

(30) $${\tilde{v}}_{j}^{n}=C_{n}^{n^{\prime }}C_{b}^{n}C_{l}^{b}(v_{j}^{l}+\delta v_{j}^{l})$$

where the perturbed attitude matrix is linearized using the small-angle approximation ${\hat{C}}_{b}^{n}\approx \left(I-\left[\delta \psi^{n}\right]\times \right)C_{b}^{n}$, with $\delta \psi^{n}$ denoting the INS attitude-error vector.

#### 3.3.2. The Landmark Position Calculated by the INS

The position vector of landmark j in the n-frame can be obtained by calculating the difference between the position of landmark j and the LiDAR’s position:

(31) $$v_{j}^{n}=r_{j}^{n}-r_{Lidar}^{n}$$

$r_{j}^{n}$ is measured in advance before navigation, while $r_{Lidar}^{n}$ represents the LiDAR position calculated by the INS, whose expression is given by Equation (32). Substituting this into Equation (31) yields:

(32) $$r_{Lidar}^{n}=r_{IMU}^{n}+C_{b}^{n}R^{b}$$

(33) $$v_{j}^{n}=r_{j}^{n}-r_{IMU}^{n}-C_{b}^{n}R^{b}$$

In Formula (33), $r_{IMU}^{n}$ denotes the inherent position of the INS itself, while $R^{b}$ represents the lever-arm error between the INS and the LiDAR, obtained through calibration procedures.

Due to position and attitude measurement errors in the INS, the calculated position vector of targets j also contains errors, expressed as follows:

(34) $${\hat{v}}_{j}^{n}=r_{j}^{n}-(r_{IMU}^{n}+\delta r_{IMU}^{n})-C_{n}^{n^{\prime }}C_{b}^{n}R^{b}$$

#### 3.3.3. Measurement Equations

In the Kalman filter, the observation vector z represents the discrepancy between the position vectors of targets j as measured by the LiDAR and as calculated by the INS, expressed as follows:

(35) $$z={\tilde{v}}_{j}^{n}-{\hat{v}}_{j}^{n}$$

Substituting the expressions for ${\tilde{v}}_{j}^{n}$ and ${\hat{v}}_{j}^{n}$ into Equation (42) yields:

(36) $$z=r_{j}^{n}-(r_{IMU}^{n}+\delta r_{IMU}^{n})-C_{n}^{n^{\prime }}C_{b}^{n}R^{b}-C_{n}^{n^{\prime }}C_{b}^{n}C_{l}^{b}(r_{j}^{l}+\delta r_{j}^{l})$$

Substituting $C_{n}^{n^{\prime }}$ into Formula (39) and expanding yields:

(37) $$z=\begin{array}{c} \left(r_{j}^{n}-r_{IMU}^{n})-C_{b}^{n}(R^{b}+C_{l}^{b}r_{j}^{l})+\left(\delta \psi \times \right)C_{b}^{n}(R^{b}+C_{l}^{b}r_{j}^{l}\right)\\ -C_{b}^{n}C_{l}^{b}\delta r_{j}^{l}+\left(\delta \psi \times \right)C_{b}^{n}C_{l}^{b}\delta r_{j}^{l}-\delta r_{IMU}^{n} \end{array}$$

Let $r_{j}^{n}-r_{IMU}^{n}$ be the true value of the landmark position vector calculated by the INS, and $C_{b}^{n}(R^{b}+C_{l}^{b}r_{j}^{l})$ be the true value of the landmark position vector jointly measured by the INS and LiDAR. Given that their magnitudes are equal, we have:

(38) $$(r_{j}^{n}-r_{IMU}^{n})-C_{b}^{n}(R^{b}+C_{l}^{b}r_{j}^{l})=0$$

The product of two first-order error quantities in the immediately preceding expansion is second order and is therefore neglected. Retaining only first-order terms gives the linearized observation relationship used by the Kalman filter.

(39) $$z=\left(\delta \psi \times \right)C_{b}^{n}(R^{b}+C_{l}^{b}r_{j}^{l})-C_{b}^{n}C_{l}^{b}\delta r_{j}^{l}-\delta r_{IMU}^{n}$$

Using the equality of the two nominal landmark vectors and collecting the remaining first-order position, attitude, lever-arm, and LiDAR measurement terms yields the observation equation below.

(40) $$z=C_{2}\delta \psi -C_{b}^{n}C_{l}^{b}\delta r_{j}^{l}-\delta r_{IMU}^{n}$$

Substituting the Cartesian LiDAR measurement perturbation from Section 3.3.1 into this linearized relationship yields the final measurement matrix H.

(41) $$z=-\delta r_{IMU}^{n}+C_{2}\delta \psi -C_{b}^{n}C_{l}^{b}C_{1}\delta d$$

(42) z=Hx+W

(43) $$H=\left[\begin{array}{cccccc} -I_{3\times 3} & 0 & C_{2} & 0 & 0 & -C_{b}^{n}C_{l}^{b}C_{1} \end{array}\right]$$

## 4. Experimental Tests

**Figure 4:**
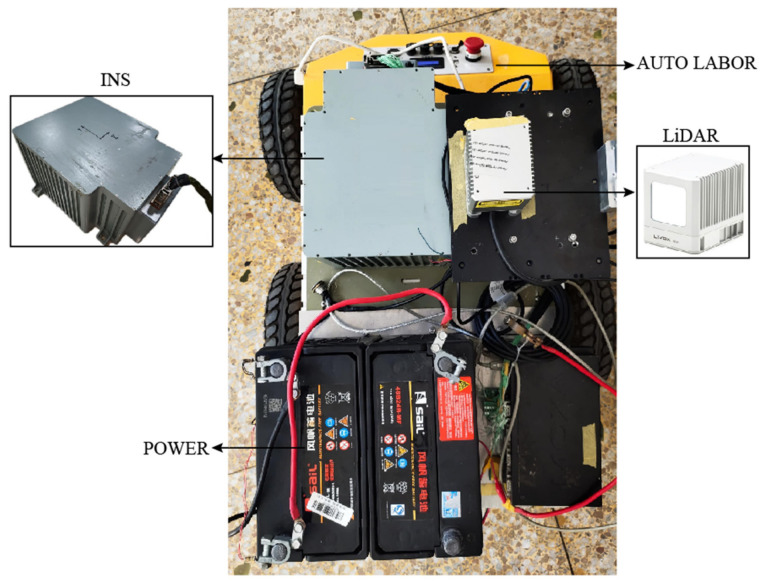
Test system.

The proposed method was evaluated through experimental testing. As shown in Figure 4, the test system comprises a FOG-based INS, LiDAR, an unmanned ground vehicle, battery power supply, and a data acquisition laptop. The FOG-INS integrates three fiber optic gyroscopes, three quartz accelerometers, and associated processing electronics. Specifications for both the FOG-INS and LiDAR are provided in Table 2.

**Figure 5:**
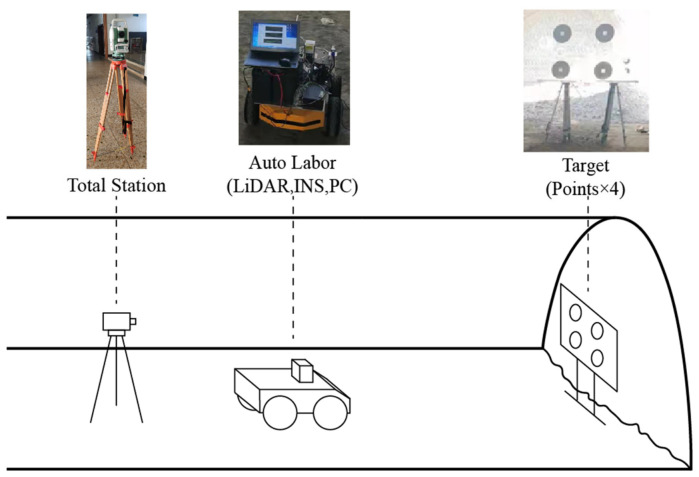
Test schematic diagram.

The full experimental setup comprises: (1) Four retroreflective fiducial targets; (2) A vehicle-mounted INS and LiDAR platform; (3) A total station (providing ground truth coordinates for LiDAR and control points); and (4) A computational workstation. The system configuration is depicted in Figure 5.

### 4.1. Experimental Setup, Calibration, and Reference Frames

The world frame used for the reported navigation solution is the local NED frame n. A total-station frame T is first established from surveyed control points. The NED origin O is selected at the center of ground control point CP1, located on the corridor centerline at the retroreflective-marker end, the north axis is defined by the surveyed baseline from CP1 to CP2, with a baseline length of 18.8m and oriented along the longitudinal axis of the corridor, the east axis completes the right-handed horizontal frame, and the down axis is opposite the local vertical/up direction. A point measured in the total-station frame is transformed as follows:

(44) $$P^{n}=R_{T}^{n}(P^{T}-P_{o}^{T})$$

where $R_{T}^{n}$ is the surveyed rotation from T to n and $P_{o}^{T}$ is the NED-origin coordinate in T. The same transformation is applied to the total-station reference points and the LiDAR–INS estimates before computing north, east, and down errors.

The LiDAR–IMU rotational and translational extrinsic parameters were calibrated before the experimental campaign and verified before each experimental run. The residual uncertainty of the calibrated relative rotation was approximately about 0.035°, and the residual lever-arm uncertainty was approximately about 3.2 mm. Before each run, the platform was maintained stationary for 180 s to initialize the INS attitude and estimate the initial gyroscope and accelerometer biases.

Static LiDAR characterization was performed before the navigation trials using fixed retroreflective targets at independently surveyed coordinates. For each distance and incidence condition, the mean bias, standard deviation, and target-center repeatability were computed before and after calibration. Table 3 statistics provide the bias corrections and $R_{k}$ used by the filter.

Position accuracy is evaluated at total-station checkpoints. For M matched estimates and reference positions, the three-dimensional RMSE is defined as follows:

$${\mathrm{RMSE}}_{3D}=\sqrt{\frac{1}{M}{\sum_{k=1}^{M}\left\|p_{k,est}^{n}-p_{k,ref}^{n}\right\|}_{2}^{2}}$$

Axis-specific RMSE values are calculated analogously from $e_{N}$, $e_{E}$, and $e_{D}$. Because the total station was used during stationary phases, the manuscript reports checkpoint accuracy rather than claiming continuous dynamic ground truth. Each experimental run contained six stationary checkpoints, and five independent runs were completed. At each checkpoint, the platform remained stationary for 5 s, and 50 LiDAR–INS position estimates were averaged. The total station had a nominal angular accuracy of 6 arcsec and a distance-measurement accuracy of ±(2 mm + 5 ppm). The temporal pairing tolerance between the averaged LiDAR–INS result and the total-station reference was 28 ms.

### 4.2. Laboratory Tests

**Figure 6:**
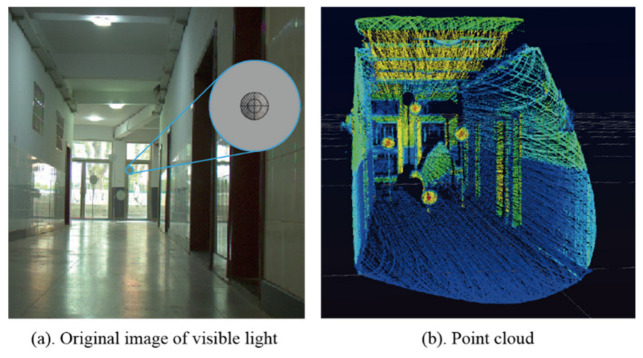
Laboratory tunnel test site image.

Laboratory experiments were conducted in the corridor shown in Figure 6. The available corridor measured approximately 30 m in length, 3 m in width, and 3.2 m in height. These dimensions were determined by the available test site rather than selected as algorithm parameters. They represent the narrow and elongated geometry of the target application and ensured that the four markers remained within the measurement range and FoV of the Livox Mid-40 throughout the trajectory.

Four circular retroreflective markers, each 0.20 m in diameter, were arranged in a rectangular pattern at one end of the corridor. The horizontal and vertical center-to-center spacings were 0.80 m and 0.60 m, respectively, and the center of the marker array was approximately 1.50 m above the floor. During each run, the LiDAR-to-marker distance increased from approximately 3.0 m to 28.0 m.

The platform traveled approximately along the longitudinal axis of the corridor away from the marker end. Its mean and maximum speeds were approximately 0.60 m/s, respectively, and the effective trajectory length was approximately 25.0 m. The corridor floor consisted of relatively smooth concrete with several construction joints and minor local unevenness. The measured root-mean-square vertical acceleration during motion was approximately 0.18 m/s^2^. Five independent experimental runs were conducted using the same trajectory, marker configuration, sensor settings, and processing parameters.

**Figure 7:**
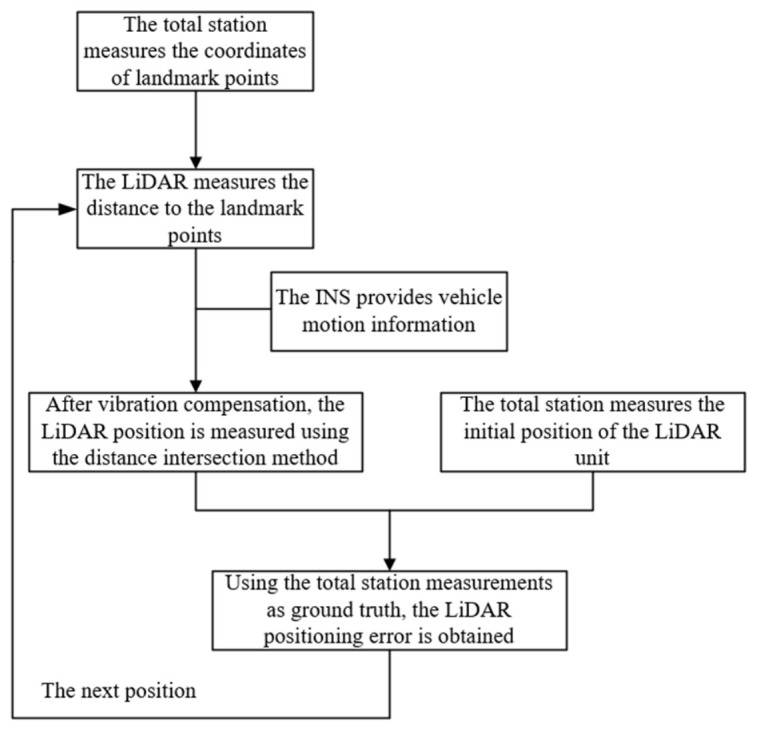
Positioning flowchart.

**Figure 8:**
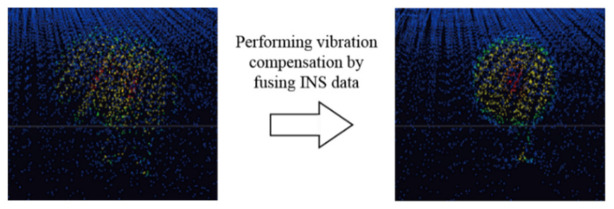
Vibration compensation result diagram.

The positioning flowchart is illustrated in Figure 7:

After the surveyed coordinates of the fiducial markers are loaded, the workstation uses the high-rate INS solution to deskew each LiDAR frame, extracts target-center observations, and updates the error-state filter. The continuously reported pose is expressed in the NED world frame defined in Section 4.1. At stationary checkpoints, the synchronized LiDAR–INS estimate is compared with the total-station reference after both are transformed to the same NED frame. Figure 8 illustrates the effect of point-wise vibration compensation on the target point cluster.

To avoid an ambiguous eigenvalue ratio, point-cloud distortion is quantified by the orthogonal target-fitting residual. Let $d_{i}$ be the shortest distance from the i-th retained point to the fitted target ellipse in the local target plane. The distortion metric is defined as

(45) $$D_{\mathrm{fit}}=\sqrt{\frac{1}{N}\sum_{i=1}^{N}d_{i}^{2}}$$

A lower $D_{\mathrm{fit}}$ indicates a more compact and geometrically consistent target cluster. The metric is computed for the same target and range before and after deskewing, and the results are reported over multiple frames as mean ± standard deviation. Five independent experimental runs were evaluated, with 500 LiDAR frames retained from each run, resulting in a total of 2500 frames. All four retroreflective targets were included, yielding 10,000 paired target-frame observations over a target-range interval of approximately 3–28 m.

Before deskewing, the mean target-fitting residual was $D_{fit}=3.38\pm 0.83\;cm$. After rotational and translational deskewing, it decreased to 1.31±0.39 cm. This corresponds to an average reduction of 2.07 cm, or 61.2%. To avoid treating temporally adjacent frames as statistically independent, the statistical comparison was performed using the mean result of each independent run. A paired-sample t-test showed that the reduction was statistically significant *p* < 0.001), with a 95% confidence interval of 1.49–2.65 cm for the mean reduction.

### 4.3. Field Tests

**Figure 9:**
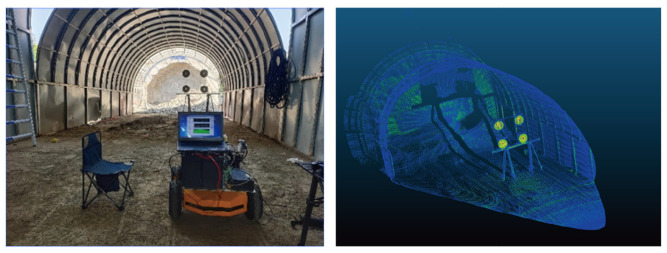
Field test diagram.

Field tests were conducted in an available construction tunnel approximately 30 m long, 4 m wide, and 3.5 m high. Four retroreflective markers were arranged near the tunneling face with horizontal and vertical center-to-center spacings of 0.9 m and 0.7 m, respectively, and the marker-array center was mounted approximately 1.6 m above the ground. During each run, the target range increased from approximately 3 m to 30 m. The platform traveled along a 26 m trajectory at a mean speed of 0.35 m/s and a maximum speed of 0.55 m/s. The tunnel floor was uneven, with a measured vertical-acceleration RMS of approximately 0.72 m/s^2^ during motion. Five independent runs were conducted using the same trajectory and marker arrangement. The experiment situation is shown in Figure 9.

**Figure 10:**
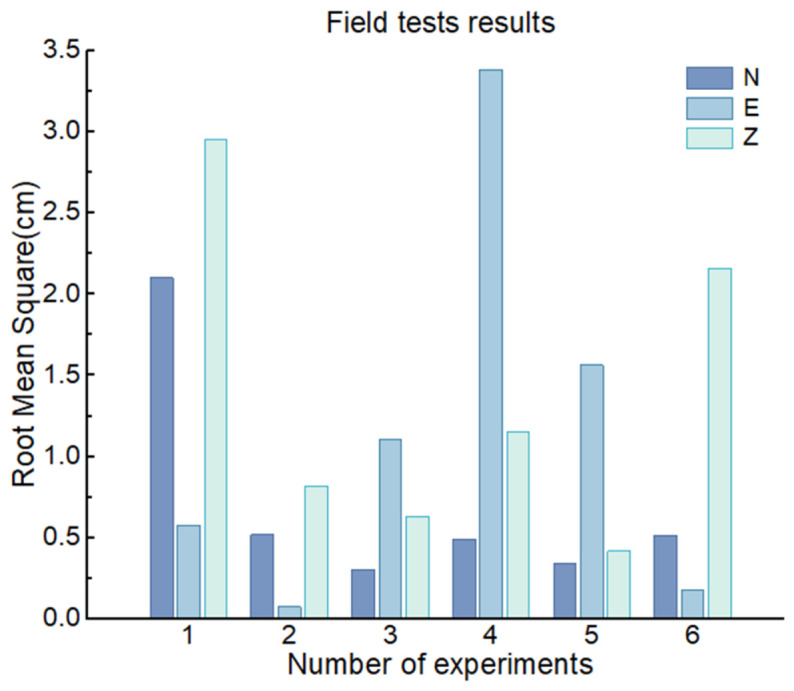
Test results.

During each of the five independent runs, the platform stopped successively at the same six checkpoints. At each checkpoint, the platform remained stationary for 5 s, and 50 consecutive LiDAR–INS position estimates acquired at 10 Hz were averaged to obtain the reported position. The total-station measurement and the midpoint timestamp of the LiDAR–INS averaging interval were paired using a maximum temporal tolerance of 28 ms. The results shown in Figure 10 are the averages over the five repeated runs at the six checkpoints.

As evidenced in the preceding figure, the real-time positioning accuracy of the system demonstrates:

- North direction: Peak RMS error of 2.10 cm with average error below 1 cm.
- East direction: Peak RMS error of 3.38 cm with average error below 2 cm.
- Down direction: Peak RMS error of 2.95 cm with average error below 2 cm.

These positioning metrics satisfy the high-precision requirements for tunnel navigation applications.

### 4.4. Ablation and Baseline Comparisons

To evaluate the contributions of the individual components of the proposed method, baseline and ablation experiments were conducted using the same tunnel trajectory, retroreflective-marker layout, sensor data, initialization procedure, and reference coordinates. The following six configurations were compared:

1. LiDAR only: marker-based positioning using the raw LiDAR observations without INS fusion or point-wise deskewing;
2. INS only: inertial navigation without LiDAR measurement updates;
3. LiDAR–INS without deskewing: LiDAR–INS fusion using the original, uncompensated LiDAR frames;
4. Rotation-only deskewing: point-wise compensation using only the interpolated INS attitude;
5. Rotation-and-translation deskewing: point-wise compensation using both the interpolated INS attitude and position;
6. Complete proposed method: rotation-and-translation deskewing combined with the offline LiDAR angular-bias calibration, LiDAR–INS temporal alignment, and error-state Kalman-filter fusion.

All configurations were evaluated using the same five repeated runs and the same six stationary checkpoints. At each checkpoint, the synchronized positioning results collected during the 5 s stationary interval were averaged before comparison with the total-station reference. The maximum error in Table 4 denotes the maximum three-dimensional position error among the evaluated checkpoint estimates. The processing time denotes the mean computation time required for one LiDAR frame on the Intel Core i7-12700H computer.

The LiDAR-only solution exhibited a three-dimensional RMSE of 12.52 cm, whereas the INS-only solution produced a substantially larger RMSE of 24.60 cm because of accumulated inertial drift. Introducing LiDAR measurement updates without point-wise deskewing reduced the three-dimensional RMSE to 9.05 cm. However, the remaining target-fitting RMSE of 3.64 cm indicates that intra-frame motion distortion continued to affect target-center estimation.

Compared with the LiDAR–INS configuration without deskewing, rotation-only deskewing reduced the target-fitting RMSE from 3.64 cm to 2.47 cm, corresponding to a reduction of 32.1%. The three-dimensional positioning RMSE decreased from 9.05 cm to 6.51 cm, representing an improvement of 28.1%. These results show that rotational motion is a major source of point-cloud distortion in the tested tunnel trajectory.

Adding translational compensation further reduced the target-fitting RMSE from 2.47 cm to 1.80 cm and the three-dimensional positioning RMSE from 6.51 cm to 5.32 cm. These reductions, amounting to 27.1% and 18.3%, respectively, demonstrate that translational motion during the LiDAR frame cannot be neglected, even though its contribution was smaller than that of rotational motion under the tested operating conditions.

The complete method achieved the lowest target-fitting RMSE, three-dimensional positioning RMSE, and maximum position error. Relative to LiDAR–INS fusion without deskewing, the complete method reduced the target-fitting RMSE by 64%, from 3.64 cm to 1.31 cm, and reduced the three-dimensional positioning RMSE by 45.4%, from 9.05 cm to 4.94 cm. The maximum three-dimensional error was also reduced from 15.2 cm to 8.4 cm. The additional improvement over rotation-and-translation deskewing reflects the combined contribution of the offline angular-bias calibration and temporal alignment.

The accuracy improvement was accompanied by an increase in computational cost. The mean processing time increased from 22.4 ms per frame without deskewing to 43.8 ms per frame for the complete method. Nevertheless, the complete processing chain remained below the 100 ms frame period of the 10 Hz LiDAR implementation. Therefore, the method satisfies the average computational-throughput requirement of the experimental system.

### 4.5. Illumination Robustness Evaluation

The illumination experiments were conducted under three conditions using the same marker arrangement, target range, platform trajectory, speed, and processing parameters. For the dark condition, all corridor lights were switched off. Normal illumination was provided by the existing ceiling-mounted LED lamps. Strong background illumination was generated using two LED floodlights directed toward the retroreflective markers. Illuminance was measured at the marker surface using a calibrated digital lux meter with an accuracy of approximately ±5%. Each condition was tested in five independent runs.

The results are summarized in Table 5. The system maintained similar performance under dark and normal lighting conditions. Under strong artificial background illumination, the valid-point ratio and target-detection success rate decreased, while the range standard deviation and positioning RMSE increased. Nevertheless, stable target detection and centimeter-level positioning were maintained.

## 5. Conclusions

This study developed an INS-aided point-wise deskewing and LiDAR–INS fusion method for retroreflective-marker-based navigation in narrow underground tunnels. High-rate INS poses were interpolated to the acquisition time of each LiDAR point to compensate for rotational and translational intra-frame distortion. Calibrated retroreflective-target observations were subsequently integrated into an error-state Kalman filter to constrain accumulated INS drift.

In the field experiments, the directional positioning RMSE values at the stationary checkpoints were 2.100 cm, 3.381 cm, and 2.953 cm in the north, east, and down directions, respectively, corresponding to a three-dimensional RMSE of 4.96 cm. Point-wise deskewing reduced the mean target-fitting residual from 3.38 ± 0.83cm to 1.31 ± 0.39cm, representing a reduction of 61.2%. The complete algorithm required an average processing time of 43.8 ms per LiDAR frame, which was below the 100 ms frame period of the 10 Hz implementation. Under dark, normal tunnel-lighting, and strong artificial-background-light conditions, the three-dimensional positioning RMSE values were 5.02 cm, 4.96 cm, and 6.18 cm, respectively. Even under strong artificial illumination, the target-detection success rate remained 94.8%, indicating that the system retained stable target recognition and centimeter-level positioning performance under the tested illumination conditions.

The proposed method nevertheless has several limitations. First, it relies on pre-deployed retroreflective markers and may lose absolute position updates when the markers are occluded or outside the LiDAR field of view. Second, the experiments were conducted in tunnels approximately 30 m long using only one LiDAR–INS hardware configuration. Third, total-station reference measurements were mainly available at stationary checkpoints rather than continuously during platform motion. In addition, the illumination tests used controlled artificial light sources and did not fully represent strong sunlight near tunnel entrances. The effects of dynamic obstacles, temperature variation, longer trajectories, different vibration spectra, and time-varying synchronization errors also require further investigation. Future work will focus on target-free or hybrid navigation, online temporal and extrinsic calibration, adaptive covariance estimation, continuous trajectory-reference acquisition, and validation in longer and more dynamic tunnel environments.

## Figures and Tables

**Table 1:** Kalman-filter covariance settings and their determination.

Value Used in Experiments	Meaning/Determination Method	Quantity
$\approx 8\times 10^{-7}\;rad/\sqrt{s}$	From gyroscope angular-random-walk specification or Allan-deviation fitting	$Q_{c}$: gyro white noise
$\approx 5\times 10^{-6}\;m^{2}/s^{3}$	From velocity-random-walk specification or Allan-deviation fitting	$Q_{c}$: accelerometer white noise
${\approx 10}^{-18}\;{rad}^{2}/s^{3}$	From bias-instability/bias-correlation analysis	$Q_{c}$: gyro-bias driving noise
${\approx 6\times 10}^{-11}\;m^{2}/s^{3}$	From bias-instability/bias-correlation analysis	$Q_{c}$: accelerometer-bias driving noise
INS: 400 Hz, LiDAR: 10 Hz	INS propagation rate/LiDAR measurement-update rate	Filter rates

**Table 2:** Sensor parameters.

Sensors	Parameters	Value
FOG	Bias Stability	0.01°/h, 1σ
	Bias Repeatability	0.01°/h,1σ
	Angular Random Walk	$0.002{}^{\circ}/\sqrt{h}$
Quartz Accelerometer	Bias Stability	20 μg,1σ
	Bias Repeatability	20 μg,1σ
	Scale Factor Stability	20 ppm
	Velocity Random Walk	$300\;\mu g/\sqrt{Hz}$
LiDAR	Detection Range	130 m (20% reflectivity)
	FoV	38.4° (circular)
	Range Precision	2 cm (nominal, 1 σ under specified conditions)
	Angular Accuracy	<0.1°

**Table 3:** Static LiDAR noise and bias characterization.

Quantity	Test Condition/Determination	Value Used
Range bias $b_{r}$	Known target distances; mean measured-minus-reference range	Original: 1.2 cm, After calibration: ≤±0.6 cm
Elevation bias $b_{\alpha }$	Surveyed target direction; mean angular residual	Original: 0.28°, After calibration: ≤±0.05°
Azimuth bias $b_{\beta }$	Surveyed target direction; mean angular residual	Original: 0.24°, After calibration: ≤±0.03°
Target-center repeatability	Standard deviation of repeated static center estimates	0.3–1.2 cm

**Table 4:** Baseline and ablation results.

Time/Frame (ms)	Max. Error (cm)	3D RMSE (cm)	N/E/D RMSE (cm)	Target-Fit RMSE (cm)	Configuration
12.8	17.5	12.52	5.3/6.2/9.5	3.65	LiDAR only
0.8	44.9	24.60	13.8/16.3/12.2	N/A	INS only
22.4	15.2	9.05	4.6/5.2/5.8	3.64	LiDAR-INS, no point-wise deskewing
28.4	11.7	6.51	3.4/4.6/3.1	2.47	Rotation-only deskewing
36.9	9.3	5.32	2.4/3.6/3.1	1.80	Rotation + translation deskewing
43.8	8.4	4.96	2.10/3.38/2.95	1.31	Complete proposed method

**Table 5:** LiDAR and navigation performance under different illumination conditions.

Condition	Illuminance (lx)	Valid-Point Ratio (%)	Detection Success (%)	Range Standard Deviation (cm)	Target-Fit RMSE (cm)	3D Positioning RMSE (cm)
Dark/lights off	0.3	98.4	99.3	1.02	1.27	5.02
Normal tunnel lighting	156	97.8	99.1	1.16	1.31	4.96
Strong artificial background light	3650	89.6	94.8	1.78	1.74	6.18

## Data Availability

The authors confirm that the data supporting the findings of this study are available within the article.
